# Prenylated Indolediketopiperazine Peroxides and Related Homologues from the Marine Sediment-Derived Fungus *Penicillium brefeldianum* SD-273

**DOI:** 10.3390/md12020746

**Published:** 2014-01-27

**Authors:** Chun-Yan An, Xiao-Ming Li, Chun-Shun Li, Gang-Ming Xu, Bin-Gui Wang

**Affiliations:** 1 Key Laboratory of Experimental Marine Biology, Institute of Oceanology, Chinese Academy of Sciences, Nanhai Road 7, Qingdao 266071, China; E-Mails: anchy5885@gmail.com (C.-Y.A.); lixmqd@yahoo.com.cn (X.-M.L.); lichunshun@ms.qdio.ac.cn (C.-S.L.); aericxu@gmail.com (G.-M.X.); 2 University of Chinese Academy of Sciences, Yuquan Road 19A, Beijing 100049, China

**Keywords:** marine sediment, *Penicillium brefeldianum* SD-273, indolediketopiperazine, brine shrimp lethality

## Abstract

Three new indolediketopiperazine peroxides, namely, 24-hydroxyverruculogen (**1**), 26-hydroxyverruculogen (**2**), and 13-*O*-prenyl-26-hydroxyverruculogen (**3**), along with four known homologues (**4**–**7**), were isolated and identified from the culture extract of the marine sediment-derived fungus *Penicillium brefeldianum* SD-273. Their structures were determined based on the extensive spectroscopic analysis and compound **1** was confirmed by X-ray crystallographic analysis. The absolute configuration of compounds **1**–**3** was determined using chiral HPLC analysis of their acidic hydrolysates. Each of the isolated compounds was evaluated for antibacterial and cytotoxic activity as well as brine shrimp (*Artemia salina*) lethality.

## 1. Introduction

**Figure 1:**
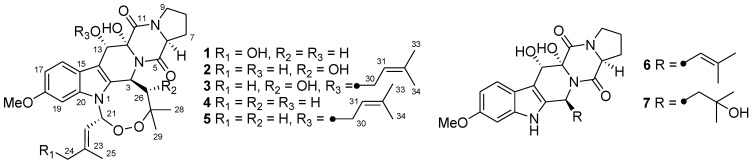
The structures of isolated compounds **1**–**7**.

Indolediketopiperazine alkaloids are a series of natural occurring secondary metabolites generally formed from l-tryptophan and other common amino acids, e.g., l-proline, l-alanine, and d-valine [1,2,3,4,5,6,7,8]. Some of these compounds showed tremor-producing, cytotoxic, antibacterial, and brine shrimp lethal activity [3,4,5,6,7]. In our continuing investigation aimed to explore new and bioactive secondary metabolites from marine-derived fungi [9,10,11,12,13,14,15], we recently focused on a fungal strain *Penicillium brefeldianum* SD-273 that was isolated from the sediment samples collected from the estuary of the Pearl River in the South China Sea. The EtOAc extract of the fermentation broth showed moderate brine shrimp (*Artemia salina*) lethality in the preliminary assays. Further investigation of the culture extracts of this fungus resulted in the isolation of three new indolediketopiperazine peroxides (**1**–**3**) and four known homologues (**4**–**7**) (Figure 1). The structure of these compounds was determined by spectroscopic analysis and compound **1** was confirmed by X-ray crystallographic experiment. The absolute configuration of compounds **1**–**3** was determined by chiral HPLC analysis of their acidic hydrolysates. It should be noted that, based on our 1D and 2D NMR data, the original assignment of the carbon signals for the two methyls in the isoprenyl moiety of verruculogen (**4**) [4] should be interchanged. This paper describes the isolation, structure determination, and bioactivity evaluation of the isolated compounds.

## 2. Results and Discussion

### 2.1. Structure Elucidation of the New Compounds **1**–**3**

The cultured broth and mycelia of the fungus were exhaustively extracted with EtOAc and MeOH, respectively. Since the HPLC and TLC profiles of the two extracts were nearly identical, they were combined before further separation. The combined extract was fractionated by a combination of column chromatography (CC) on Si gel, reversed-phase Si gel C18, and Sephadex LH-20 as well as by semi-preparative HPLC and preparative thin layer chromatography (pTLC), to yield seven indolediketopiperazine derivatives (**1**–**7**) (Figure 1).

**Figure 2:**
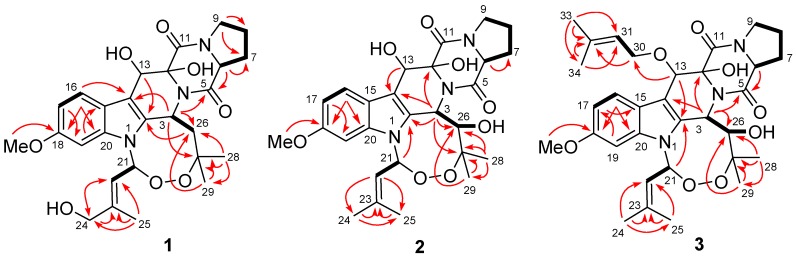
Key HMBC (red arrows) and ^1^H–^1^H COSY (bold line) correlations of **1**–**3**.

**Figure 3:**
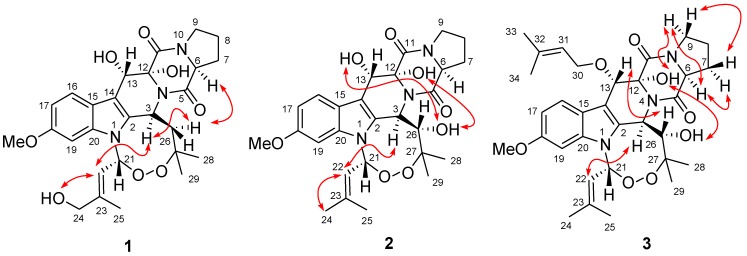
Key NOE correlations (double arrows) of **1**–**3**.

Compound **1** was obtained as colorless crystals. The HRESIMS experiment established its molecular formula C_27_H_33_N_3_O_8_. Inspection of the ^1^H-, ^13^C-, and DEPT NMR data of **1** revealed the presence of four methyls (with one *O*-methyl group), five sp^3^ methylenes (with one oxygenated), four sp^3^ and four sp^2^ methines, and ten quaternary carbons (with two oxygenated sp^3^ and six sp^2^ carbons and two ester/amide carbons), as well as three exchangeable protons (Table 1). The ^1^H- and ^13^C-NMR data assignments matched well with those of the corresponding signals for verruculogen (**4**), a tremorgenic mycotoxin peroxide isolated from peanuts-derived fungus *Penicillium verruculosum* [4,5], except for the presence of the C-24 hydroxy group, which was consistent with the difference in the molecular formula. This difference was supported by the fact that the NMR signals for one of the two methyls in the prenyl moiety of **4** [4,5] were replaced by the downfield oxygenated CH_2_ signals at δ_H_ 3.80/δ_C_ 65.2 (CH_2_-24) in the NMR spectra of **1** (Table 1). The HMBC correlations from H-22 to C-24 and C-25, from H_2_-24 to C-22 and C-23, and from H_3_-25 to C-22, C-23, and C-24 verified the above deduction (Figure 2). The observed NOE correlations from H-26α to H-3 and H-6 and from H-3 to H-22 indicated that these protons located on the same face of the molecule (Figure 3). In addition, NOE correlation from H-22 to the proton of 24-OH established the *E*-geometry for the double bond at C-22. However, the relative configuration for 12-OH and 13-OH was not assigned since no diagnostic NOE correlations could be detected.

**Table 1:** ^1^H and ^13^C NMR data of compounds **1**–**3** ^a^.

No.	1		2		3	
	δ_H_ (*J* in Hz)	δ_C_	δ_H_ (*J* in Hz)	δ_C_	δ_H_ (*J* in Hz)	δ_C_
2		131.4, C		129.6, C		129.8, C
3	5.89, d (10.0)	48.3, CH	6.13, d (8.8)	49.1, CH	6.20, d (9.0)	48.4, CH
5		171.1, C		170.7, C		171.4, C
6	4.45, t (8.0)	58.8, CH	4.35, t (7.9)	58.7, CH	4.45, t (8.2)	58.9, CH
7	α 2.30, m β 1.88, m	29.3, CH_2_	α 2.33, m β 1.90, m	29.5, CH_2_	α 2.30, m β 1.87, m	29.2, CH_2_
8	1.94, m	22.6, CH_2_	1.90, m	22.1, CH_2_	1.87, m	22.5, CH_2_
9	α 3.44, m β 3.50, t, 8.2	45.5, CH_2_	α 3.44, m β 3.52, m	44.8, CH_2_	α 3.40, m β 3.52, m	45.2, CH_2_
11		166.5, C		166.0, C		165.5, C
12		82.1, C		82.6, C		84.1, C
13	5.40, s	68.4, CH	5.43, s	68.1, CH	5.12, s	73.5, CH
14		107.9, C		107.8, C		107.5, C
15		121.3, C		120.6, C		120.4, C
16	7.72, d (8.7)	121.8, CH	7.72, d (9.3)	121.1, CH	7.57, d (8.7)	120.3, CH
17	6.69, dd (8.7, 1.3)	109.3, CH	6.69, dd (9.3, 2.1)	108.7, CH	6.74, dd (8.7, 2.2)	108.8, CH
18		156.0, C		155.3, C		155.3, C
19	6.75, d (1.3)	94.3, CH	6.70, d (2.1)	93.4, CH	6.69, d (2.2)	93.6, CH
20		136.3, C		135.3, C		135.3, C
21	6.88, d (8.4)	85.5, CH	6.78, d (8.2)	85.0, CH	6.77, d (8.2)	85.1, CH
22	5.26, d (8.4)	116.0, CH	5.06, d (8.2)	118.3, CH	5.02, d (8.2)	118.1, CH
23		146.9, C		142.8, C		143.0, C
24	3.80, s	65.2 CH_2_	1.73, s	25.2, CH_3_	1.74, s	25.4, CH_3_
25	1.87, s	14.5, CH_3_	2.00, s	18.3, CH_3_	2.00, s	18.4, CH_3_
26	α 1.91, m β 1.55, m	51.3, CH_2_	2.92, d (8.8)	80.0, CH	2.92, d (9.0)	79.3, CH
27		83.2, C		85.0, C		85.2, C
28	1.57, s	24.6, CH_3_	1.44, s	18.4, CH_3_	1.41, s	18.3, CH_3_
29	0.95, s	27.2, CH_3_	0.99, s	25.3, CH_3_	0.98, s	25.2, CH_3_
30					4.56, dd (7.0, 11.0) 4.88, dd (6.5, 11.0)	68.5, CH_2_
31					5.57, t (6.5)	122.2, CH
32						134.5, C
33					1.75, s	18.1, CH_3_
34					1.77, s	25.2, CH_3_
18-OCH_3_	3.75, s	55.8, CH_3_	3.76, s	55.2, CH_3_	3.76, s	55.2, CH_3_
12-OH	5.10, br s ^b^		5.26, br s ^b^		6.16, br s	
13-OH	6.68, br s ^b^		6.32, br s ^b^			
24-OH	3.34, br s					
26-OH			5.52, br s		5.37, br s	

^a^ Measured in DMSO-*d*_6_, at 500 MHz for ^1^H and 125 MHz for ^13^C with reference to the solvent signals, δ in ppm; ^b^ Exchangeable signals in the same column.

**Figure 4:**
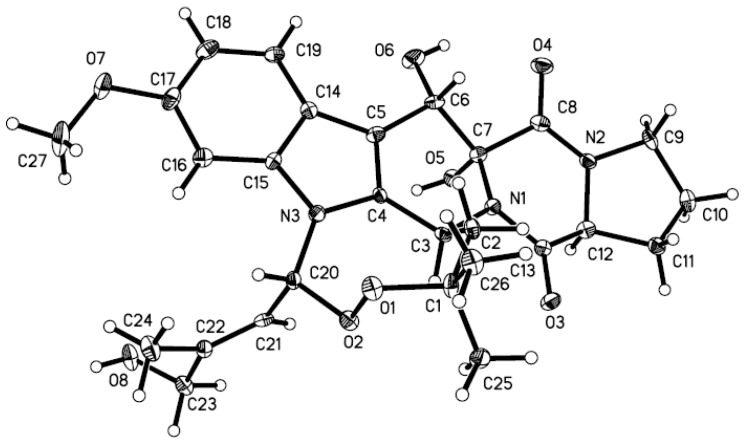
X-ray structure of compound **1**. (Note: A different numbering system is used for the structure in the text.)

To unambiguously assign the structure, a single X-ray diffraction study was performed. However, compound **1** was difficult to get suitable crystal for X-ray analysis and after many attempts, X-ray quality crystals were obtained by slow evaporation of a solution of **1** in CHCl_3_-MeOH (1:1). The X-ray diffraction crystallographic analysis confirmed the structure and relative configuration for **1** (Figure 4).

**Figure 5:**
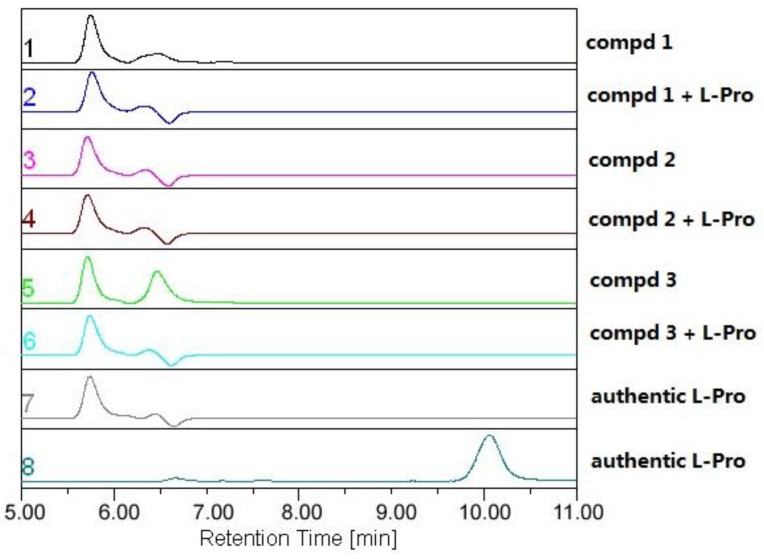
Chiral HPLC analysis of the amino acid derivatives of compounds **1**–**3** (Phenomenex-Chirex 3126 column, 250 × 4.60 mm, 5 μm; Gradient: 10% aqueous acetonitrile with 2 mM CuSO_4_·5H_2_O, detected at 225 nm).

The absolute configuration of **1** was deduced after chiral HPLC analysis of the degradation products of the acidic hydrolysate. The HPLC profile of the hydrolysate was compared with authentic standard to establish the configuration of the amino acid-derived unit as l-proline (Figure 5), corresponding to the *S*-configuration for C-6. The absolute configuration of compound **1** was therefore assigned to be 3*S*, 12*R*, 13*S*, and 21*R*. The structure for compound **1** was finally determined and it was named as 24-hydroxyverruculogen.

Compound **2** was isolated as a pale amorphous solid. Its molecular formula was demonstrated as C_27_H_33_N_3_O_8_ by HRESIMS data. Analysis of its ^1^H and ^13^C NMR spectral data (Table 1) with those of verruculogen (**4**) indicated that both compounds shared the same indolediketopiperazine skeleton [4,5]. The main difference between the two compounds was the methylene carbon resonating at δ_C_ 45.3 (C-26, this carbon was numbered as C-19 in the reference 4) in the ^13^C NMR spectrum of **4** [4] being replaced by an oxygenated methine (δ_C_ 80.0, C-26) in that of **2**. This difference was verified by the ^1^H–^1^H COSY correlations from oxymethine proton H-26 to H-3 (δ_H_ 6.13, d, *J* = 8.8 Hz) and to the exchangeable proton signal at δ_H_ 5.52 (br s, 26-OH). The observed HMBC correlations from H-3, H_3_-28, and H_3_-29 to C-26 (Figure 2) supported this deduction. Other ^1^H–^1^H COSY and HMBC correlations confirmed the structure of **2** (Figure 2). However, the remaining two exchangeable protons resonating at δ_H_ 5.26 and 6.32, which belonging to 12-OH and 13-OH, could not be unambiguously ascribed since they didn’t show any correlations in the 2D NMR spectra.

The relative configuration of compound **2** was determined by NOESY experiment, proton coupling constant, and NMR data comparison. The *trans*-relationship between H-3 and H-26 was deduced on the basis of large coupling constant (*J*_3,26_ = 8.8 Hz) and by the fact that there is no NOE correlation could be detected among them in the NOESY spectrum. The NOE correlations from the proton of 26-OH to the protons of 12-OH and 13-OH implied the same direction of these OH groups, while the observed NOE correlation from H-3 to the olefinic H-22 indicated the same orientation of H-3 and CH-22 (Figure 2). Unfortunately, the NOESY data for compound **2** proved to be insufficient to solve the relative configuration at C-6. However, based on the fact of the very similar NMR data of H-6 to H-9 and of C-6 to C-9 (Table 1) in **1** and **2**, as well as from the biogenetic consideration, the configuration of H-6 was assigned as α-orientation, the same as that of compound **1**.

The absolute configuration of **2** was also determined by amino acid analysis of its acidic hydrolysate (Figure 5), which assigned *S*-configuration for C-6. The other chiral centers in **2** were therefore deduced as 3*R*, 12*R*, 13*S*, 21*R*, and 26*S*. The structure for compound **2** was thus determined and it was named as 26-hydroxyverruculogen.

Compound **3** was also isolated as a pale amorphous solid. Its molecular formula was determined to be C_32_H_41_N_3_O_8_ on the basis of HRESIMS data. Detailed comparison of the 1D and 2D NMR data of **3** with those of **2** revealed the same structural features, except for the presence of five additional carbon signals at δ_C_ 68.5 (CH_2_, C-30), 122.2 (CH, C-31), 134.5 (C, C-32), 18.1 (CH_3_, C-33), and 25.2 (CH_3_, C-34) in the ^13^C NMR spectrum of **3**, which ascribable to the presence of a prenyl moiety in **3**. The ^1^H NMR spectrum displayed the corresponding signals for the prenyl motif at δ_H_ 4.56/4.88 (H_2_-30), 5.57 (H-31), 1.75 (H_3_-33), and 1.77 (H_3_-34). The attachment of the prenyl unit at C-13 was evidenced by the observed HMBC correlation from H-13 to the oxygenated carbon C-30 (Figure 2).

The relative configuration of compound **3** was determined by proton coupling constant and NOESY spectrum. The large coupling constant (9.0 Hz) of H-3 and H-26 (Table 1) indicated that the two protons located on the different face of the molecule. The observed NOE correlations from 12-OH to 26-OH and H-9α and from H-9α to H-6 suggested the same orientation of these groups (Figure 3), while the correlations from H-3 to H-22 and from H-26 to H-13 indicated that H-3, H-22, and 13-*O*-isoprenyl units located on the same face with 26-OH. The absolute configuration of C-6 was also determined as *S* by chiral HPLC analysis of the hydralates and the absolute configuration of compound **3** was thus established as 3*R*, 6*S*, 12*R*, 13*S*, 21*R*, and 26*S*, same as that of **2**. Compound **3** was named as 13-*O*-prenyl-26-hydroxyverruculogen.

In addition to the new compounds **1**–**3**, four known homologues verruculogen (**4**) [4,5], fumitremorgin A (**5**) [2], cyclotryprostatin A (**6**) [3], and TR-2 (**7**) [4], were also isolated and identified from the culture extracts of *P. brefeldianum* SD-273.

### 2.2. Biological Activities of the Isolated Compounds

The isolated compounds **1**–**7** were examined for antibacterial, cytotoxicity, and brine shrimp lethality. None of them showed potent antibacterial activity against two bacteria (*Escherichia coli* and *Staphyloccocus aureus*), or cytotoxicity against eight tumor cell lines (B16, HuH-7, SW-1990, Hela, Du145, H460, MCF-7, and SGC-7901). However, compound **3** showed potent lethality against brine shrimp (*Artemia salina*), with LD_50_ value of 9.44 μΜ, while the positive control colchicine had the LD_50_ value of 99.0 μΜ, and the activity might be related to the prenyl substitution at 13-OH.

## 3. Experimental Section

### 3.1. General

The melting point was determined with an SGW X-4 micro-melting-point apparatus. The optical rotations were measured on an Optical Activity A-55 polarimeter. UV data were obtained on a Lengguang Gold S54 spectrophotometer. The ^1^H, ^13^C, and 2D NMR spectroscopic data were recorded on Bruker Advance 500 spectrometer. Mass spectra were measured on a VG Autospec 3000 mass spectrometer. HPLC analysis was performed using a Dionex HPLC System equipped with a P680 pump, an ASI-100 automated sample injector, a TCC-100 column oven, a UV-DAD 340U detector, and a Dionex Acclaim ODS column (4.6 × 250 mm, 5 μm). Semi-preparative HPLC was operated using a Dionex UltiMate U3000 system using an Agilent Prep RP-18 column (21.2 × 250 mm, 10 μm) with UV detection. A Phenomenex-Chirex 3126 *N*,*S*-dioctyl-(d)-penicillamine column (250 × 4.60 mm, 5 μm) was used for chiral HPLC analysis. Column chromatography (CC) was performed with silica gel (200–300 mesh, Qingdao Marine Chemical Factory, Qingdao, Shandong, China), Lobar LiChroprep RP-18 (40–63 μm; Merck, Darmstadt, Germany), and Sephadex LH-20 (18–110 μm, Merck, Darmstadt, Germany).

### 3.2. Fungal Material

The fungus *Penicillium brefeldianum* SD-273 was isolated from a sediment sample collected from the estuary of the Pearl River in South China Sea at a depth of 100 m, in October 2010. The fungal strain grew fast on potato dextrose agar plate and the pale yellow mycelia with few spores could be observed in about 3 days at 28 °C. Fungal identification was carried out using a molecular biological protocol by DNA amplification and sequencing of the ITS region as well as by calmodulin (cmd) sequencing as described previously [16]. The sequence data derived from the fungal strain have been submitted to and deposited at GenBank under accession no. JQ306332 (ITS) and KJ160447 (cmd). A BLAST search result showed that the ITS rDNA sequence was same (100%) to the sequence of *Eupenicillium brefeldianum* B37 (GenBank accession no. EF488446. It should be noted that the genus *Eupenicilium* is not used anymore and was re-defined to belong in *Penicillium* [17]), while the calmodulin sequence of the strain SD-273 was similar (99%) to that of *Eupenicillium brefeldianum* AS3.6689 (accession no. AY678593). The strain is preserved at China General Microbiological Culture Collection Center, CGMCC (Culture Collection Number CGMCC 7.160).

### 3.3. Fermentation

For chemical investigation, the fungal strain was statically cultivated in liquid potato-dextrose broth medium (1000 mL seawater, 20 g glucose, 5 g peptone, 3 g yeast extract, pH 6.5–7.0, liquid medium/flask = 300 mL) in 1 L Erlenmeyer flasks for 30 days at room temperature.

### 3.4. Extraction and Isolation

The fermented whole broth (300 mL × 100 flasks) was filtered through cheesecloth to separate the culture broth and mycelia, which were extracted with EtOAc and MeOH, respectively. The two extracts were combined for further separation since their HPLC and TLC profiles were almost identical. The combined extracts (45.1 g) were subjected to Si gel vacuum liquid chromatography (VLC) using CHCl_3_–MeOH gradient elution to get eight fractions (Frs.1–8). Fr.7 (5.0 g) was subjected to column chromatography (CC) on silica gel eluted with petroleum ether–EtOAc (from 20:1 to 1:1) to give eight subfractions (Frs.7.1–7.8). Fr.7.6 (67.0 mg) was further purified by CC on Sephadex LH-20 (MeOH) and preparative TLC to obtain compound **5** (17.9 mg). Fr.8 (6.3 g) was further separated by CC on reversed phase silica gel C18 with a MeOH–H_2_O gradient (from 20% to 100%) to get nine subfractions (Frs.8.1–8.9). Fr.8.5 (410.0 mg) was subjected to semi-preparative HPLC (Dionex UltiMate U3000, 21.2 × 250 mm, 10 μm, 85% aqueous MeOH, 16 mL/min) to afford compounds **6** (99.0 mg, *t*_R_ 10.2 min) and **4** (44.0 mg, *t*_R_ 12.1 min). Fr.8.7 (230.0 mg) was purified by preparative TLC (CHCl_3_–MeOH, 15:1) to yield compounds **1** (16.0 mg) and **2** (14.0 mg). Fr.8.9 (224.0 mg) was further subjected to CC on Sephadex LH-20 to obtain compounds **3** (8.6 mg) and **7** (5.4 mg).

24-Hydroxyverruculogen (**1**): colorless crystals (CH_3_Cl–MeOH, 1:1); mp 246–248 °C; 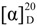 −12.5 (*c* 0.24, MeOH); UV (MeOH) λ_max_ (log ε) 223 (4.51), 268 (3.70), 288 (3.64) nm; ^1^H and ^13^C NMR data, see Table 1; ESIMS *m/z* 550 [M + Na]^+^; HRESIMS *m/z* 550.2153 [M + Na]^+^ (calcd for C_27_H_33_N_3_O_8_Na^+^, 550.2165, Δ −1.2 mmu).

26-Hydroxyverruculogen (**2**): pale amorphous solid (MeOH); 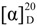 −75.5 (*c* 0.23, MeOH); UV (MeOH) λ_max_ (log ε) 223 (4.45), 271 (3.59), 287 (3.54) nm; ^1^H and ^13^C NMR data, see Table 1; ESIMS *m/z* 550 [M + Na]^+^; HRESIMS *m/z* 550.2156 [M + Na]^+^ (calcd for C_27_H_33_N_3_O_8_Na^+^, 550.2165, Δ −0.9 mmu).

13-*O*-Prenyl-26-hydroxyverruculogen (**3**): pale amorphous solid (MeOH); 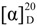 −10.1 (*c* 0.35, MeOH); UV (MeOH) λ_max_ (log ε) 227 (4.60), 275 (3.88), 289 (3.80) nm; ^1^H and ^13^C NMR data, see Table 1; ESIMS *m/z* 618 [M + Na]^+^; HRESIMS *m/z* 618.2804 [M + Na]^+^ (calcd for C_32_H_41_N_3_O_8_Na^+^, 618.2791, Δ +1.3 mmu).

### 3.5. X-ray Crystallographic Analysis of Compound **1**

All crystallographic data [18] were collected on a Bruker Smart-1000 CCD diffractometer equipped with graphite-monochromatic Mo Kα radiation (λ = 0.71073 Å) at 293(2) K. The data were corrected for absorption by using the program SADABS [19]. The structure was solved by direct methods with the SHELXTL software package [20]. All non-hydrogen atoms were refined anisotropically. The H atoms were located by geometrical calculations, and their positions and thermal parameters were fixed during the structure refinement. The structure was refined by full-matrix least-squares techniques [21].

Crystal data for **1**: C_27_H_33_N_3_O_8_; F.W. = 527.56; orthorhombic space group P2(1)2(1)2(1); unit cell dimensions *a* = 11.0113(9) Å, *b* = 13.2224(8) Å, *c* = 17.6195(7) Å, *V* = 2565.3(3) Å^3^, α = β = γ = 90°, *Z* = 4, *d*_calcd_ = 1.366 mg/m^3^, crystal dimensions 0.35 × 0.18 × 0.12 mm, μ = 0.101 mm^−^^1^, *F*(000) = 1120. The 6266 measurements yielded 4426 independent reflections after equivalent data were averaged, and Lorentz and polarization corrections were applied. The final refinement gave *R*_1_ = 0.0604 and w*R*_2_ = 0.0972 [*I* > 2σ(*I*)].

### 3.6. Amino Acid Analysis

Compounds **1** (1.0 mg), **2** (0.9 mg), and **3** (0.8 mg) were each dissolved in 10 mL 6 N HCl and heated at 110 °C for 24 h in sealed tubes. The solutions were then evaporated to dryness under reduced pressure. Each sample, including the standard amino acids l-pro and d-pro, was dissolved in 1 mL of eluting solvent (2 mM CuSO_4_·5H_2_O, with 5 mL CH_3_CN in every 100 mL solvent) and was centrifuged at a speed of 12,000 rpm to get corresponding supernate for chiral HPLC analysis, which was carried out using a Phenomenex-Chirex-3126 column at 254 nm with flow rate 1.0 mL/min at 40 °C. The HPLC analysis showed that the products of acidic hydrolysis of **1**–**3** contained the identical amino acids, which have the same retention time as that of the standard l-pro. The results established the chiral center of the proline moiety in the structure of the three compounds to be *S*-configuration (Figure 5).

### 3.7. Brine Shrimp Toxicity

Brine shrimp (*A rtemia salina*) toxicity of the isolated compounds was determined as described previously [22]. Colchicine was used as a positive control.

### 3.8. Cytotoxicity Assay

The cytotoxic activity against B16 (murine melanoma), HuH-7 (hepatocarcinoma), SW-1990 (human pancreatic adenocarcinoma), Hela (human epithelial carcinoma), Du145 (human prostate carcinoma), H460 (non-small cell lung cancer), MCF-7 (human breast adenocarcinoma), and SGC-7901 (human gastric cancer) cell lines were determined according to previously reported methods [23].

### 3.9. Antibacterial Assay

Antibacterial assay against *E. coli* and *S. aureus* was carried out using the well diffusion method [24]. Chloromycetin was used as a positive control.

## 4. Conclusions

In summary, three new indolediketopiperazine derivatives (**1**–**3**) as well as four known related homologues (**4**–**7**) were isolated and identified from the culture extract of the marine sediment-derived fungus *P. brefeldianum* SD-273 and the structures and absolute configuration were determined based on the extensive interpretation of spectroscopic data, X-ray crystallographic diffraction, and chiral HPLC analysis. Although none of these compounds showed potent antibacterial activity or cytotoxicity, compound **3** showed potent lethal activity against brine shrimp.
